# Optimized delivery of siRNA into 3D tumor spheroid cultures *in situ*

**DOI:** 10.1038/s41598-018-26253-3

**Published:** 2018-05-21

**Authors:** R. G. Morgan, A. C. Chambers, D. N. Legge, S. J. Coles, A. Greenhough, A. C. Williams

**Affiliations:** 1 School of Cellular and Molecular Medicine, University of Bristol, Biomedical Sciences Building, University Walk, Bristol, BS8 1TD UK; 2 School of Life Sciences, University of Sussex, Falmer, Brighton, BN1 9QG UK; 3 Institute of Science and the Environment, University of Worcester, Worcester, WR2 6AJ UK

## Abstract

3D tissue culture provides a physiologically relevant and genetically tractable system for studying normal and malignant human tissues. Despite this, gene-silencing studies using siRNA has proved difficult. In this study, we have identified a cause for why traditional siRNA transfection techniques are ineffective in eliciting gene silencing *in situ* within 3D cultures and proposed a simple method for significantly enhancing siRNA entry into spheroids/organoids. In 2D cell culture, the efficiency of gene silencing is significantly reduced when siRNA complexes are prepared in the presence of serum. Surprisingly, in both 3D tumour spheroids and primary murine organoids, the presence of serum during siRNA preparation rapidly promotes entry and internalization of Cy3-labelled siRNA in under 2 hours. Conversely, siRNA prepared in traditional low-serum transfection media fails to gain matrigel or spheroid/organoid entry. Direct measurement of *CTNNB1* mRNA (encoding β-catenin) from transfected tumour spheroids confirmed a transient but significant knockdown of β-catenin when siRNA:liposome complexes were formed with serum, but not when prepared in the presence of reduced-serum media (Opti-MEM). Our studies suggest a simple modification to standard lipid-based transfection protocols facilitates rapid siRNA entry and transient gene repression, providing a platform for researchers to improve siRNA efficiency in established 3D cultures.

## Introduction

Three-dimensional (3D) cell culture using primary organoids or tumour spheroids is becoming increasingly popular for modelling both normal and malignant tissue owing to its tractability, relative low cost (versus *in vivo* modelling) and increased physiological relevance^1^. Despite the rapid and widespread uptake of this technique, gene modulation methods using non-viral transfection methods *in situ* have proven technically challenging^2–5^.

Stable and long-term target gene silencing in 3D organoid culture has required shRNA, CRISPR/Cas9 or inducible knockout systems. Well established protocols for adeno-^6^ retro-^7^ and lentiviral^8^ transduction of 3D cultures already exist and CRISPR/Cas9 editing techniques have also proven successful^9,10^. However, experimental design may demand only a transient silencing of target genes, for which siRNA has been enormously successful in traditional 2D culture. siRNAs remain popular because they are simple, convenient, well-established, economical and routinely used for high-throughput screening. Previous attempts using siRNA in 3D cell culture have involved mechanical disruption of established organoids in order to obtain the single-cell suspension necessary for transfection, followed by reseeding into matrigel^11^. Another approach has involved mixing siRNA directly into the matrigel used for seeding human embryonic stem cells into 3D culture^12^. Although these approaches have shown some success, to our knowledge, there are no reports of *in situ* siRNA delivery to 3D organoids/spheroids using lipofection-based techniques despite the existence of multiple commercial products.

We report here that siRNA prepared with traditional reduced-serum transfection media (Opti-MEM) are excluded at the matrigel boundary with limited spheroid/organoid internalization. Instead, siRNA formed and delivered using standard serum-containing culture medium gains rapid entry to both matrigel and spheroids/organoids, and elicits a transient gene knockdown.

## Results

### Uptake of siRNA into tumour spheroids/primary organoids is enhanced by the presence of serum

**Figure 1:**
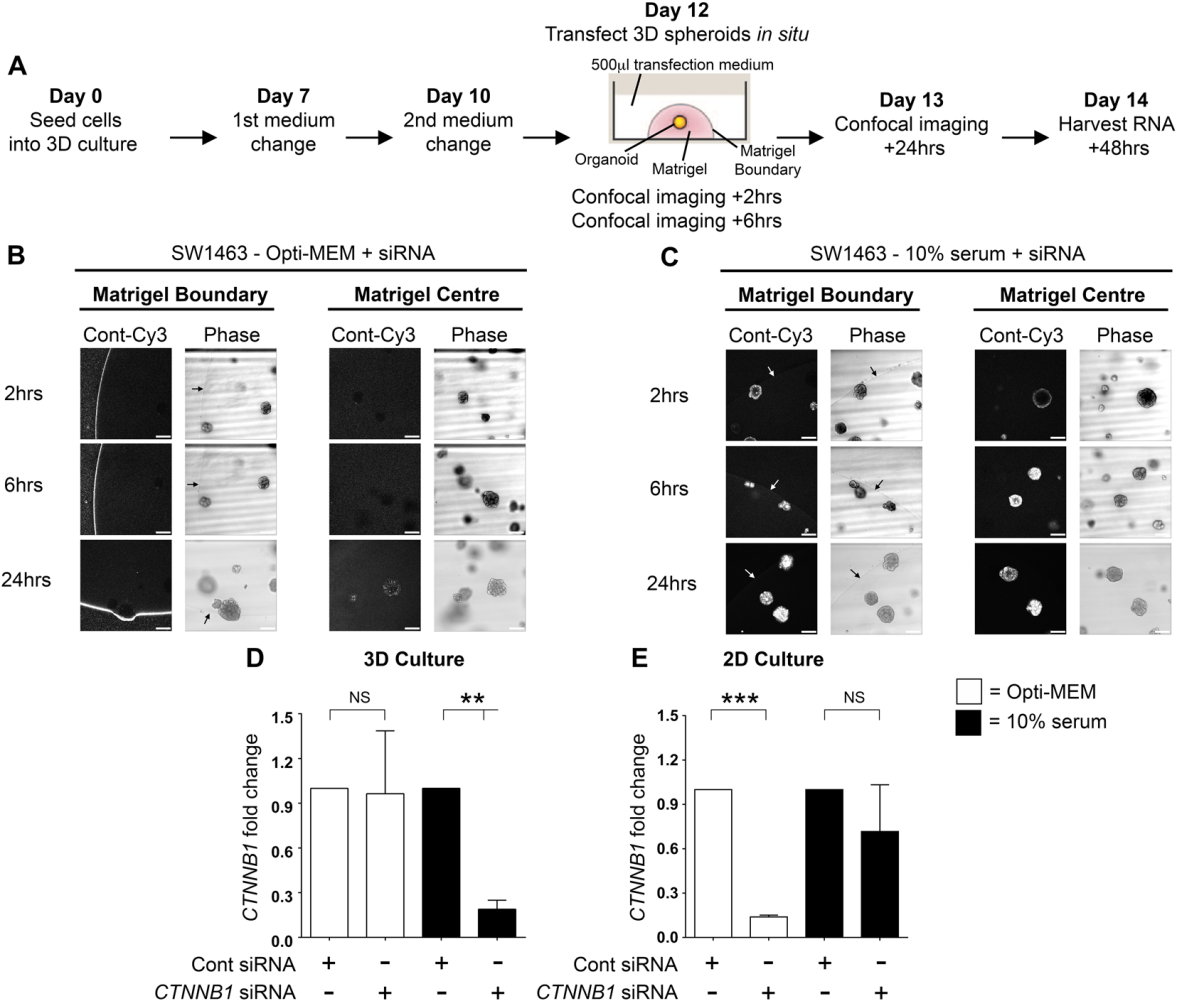
Spheroid uptake of siRNA is more efficient when complexes are formed with serum. (**A**) Workflow of experimental timeframe for spheroid transfection and analysis. (**B**) Representative confocal images of SW1463 spheroids showing localization of control-Cy3 siRNA formed in Opti-MEM using RNAiMax at 2, 6 and 24 hours post transfection. (**C**) Representative confocal images of SW1463 spheroids showing localization of control-Cy3 siRNA formed in 10% serum using RNAiMax at 2, 6 and 24 hours post transfection. White scale bar indicates 250 μM and white/black arrows indicate the location of the matrigel boundary. Summary graphs indicating the level of *CTNNB1* mRNA in (**D**) 3D, and (**E**) 2D SW1463 cell cultures, at 48 hours post transfection with control or *CTNNB1* siRNA complexes formed in Opti-MEM or 10% serum using RNAiMax. Levels are normalized to the housekeeping gene *TATA-binding protein* (*TBP*). Data represents mean ± 1 s.d (*n* = 3). Statistical significance is denoted by **P < 0.01 and ***P < 0.0001 (NS = not significant) as analyzed by a one sample t-test.

To assess the efficiency of siRNA uptake by SW1463 3D tumour spheroids we used a fluorescent Cy3-conjugated non-targeting control siRNA and formed complexes with Lipofectamine® RNAiMAX in either Opti-MEM, or DMEM containing 10% serum. The experimental timeframe is briefly summarized in Fig. 1A. Confocal imaging revealed that at 2 and 6-hour time points, siRNA complexes formed in Opti-MEM were almost completely excluded from spheroids and retained at the matrigel boundary (Fig. 1B). By 24 hours, some low-level fluorescence was detected in spheroids but the majority of fluorescence remained concentrated at the matrigel periphery. Surprisingly, the siRNA prepared with 10% serum had completely penetrated the matrigel and was internalized by spheroids in less than 2 hours (Fig. 1C). At the 6 and 24-hour time points the fluorescence level intensified within spheroids with no evidence of fluorescent accumulation at the matrigel periphery.

**Figure 2:**
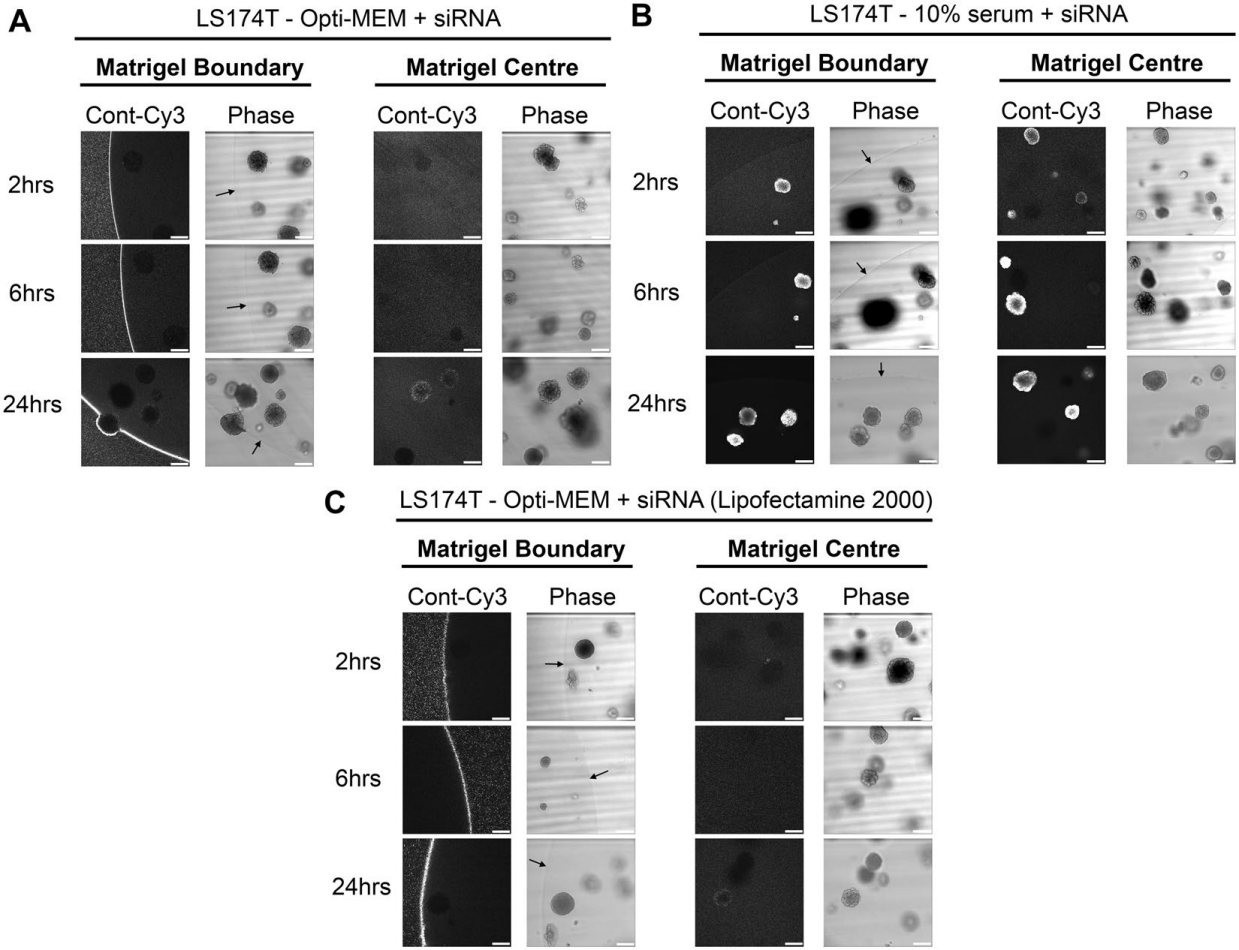
Failure of Opti-MEM prepared siRNA to penetrate matrigel or organoids is not cell line or transfection reagent specific. (**A**) Representative confocal images of LS174T spheroids showing localization of control-Cy3 siRNA formed in Opti-MEM using RNAiMax at 2, 6 and 24 hours post transfection. (**B**) Representative confocal images of LS174T spheroids showing localization of control-Cy3 siRNA formed in 10% serum using RNAiMax at 2, 6 and 24 hours post transfection. (**C**) Representative confocal images of LS174T spheroids showing localization of control-Cy3 siRNA formed in Opti-MEM using Lipofectamine 2000 at 2, 6 and 24 hours post transfection.

**Figure 3:**
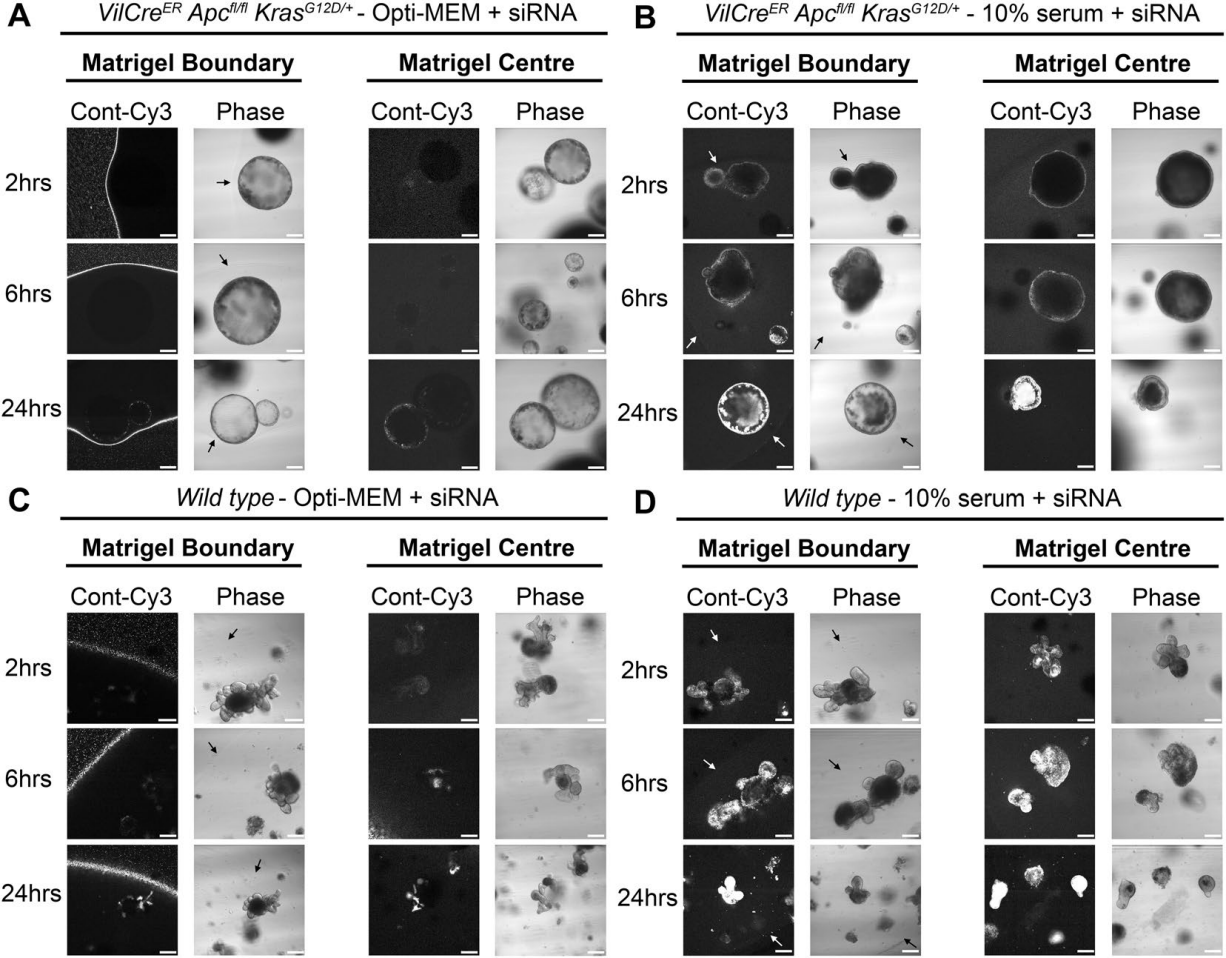
Primary organoid uptake of siRNA is more efficient when complexes are formed with serum. Representative confocal images of *VilCre*^*ER*^ *Apc*^*fl/fl*^ *Kras*^*G12D/+*^ primary mouse organoids showing localization of control-Cy3 siRNA 2, 6 and 24 hours post transfection with siRNA formed in (**A**) Opti-MEM or (**B**) 10% serum using RNAiMax. White scale bars indicate 150 μM. Representative confocal images of primary *wild type* mouse organoids showing localization of control-Cy3 siRNA 2, 6 and 24 hours post transfection with siRNA formed in (**C**) Opti-MEM or (**D**) 10% serum using RNAiMax. White scale bar indicates 80 μM except for 24 hours where it indicates 130 μM. White/black arrows indicate the location of the matrigel boundary.

To assess whether fluorescence observations translated into gene knockdown, we used a non-fluorescent siRNA targeted to *CTNNB1* under the same conditions and assessed mRNA levels direct from 3D culture using qRT-PCR. In agreement with fluorescence observations, Fig. 1D demonstrates that siRNA prepared in 10% serum exhibited a significantly more efficient *CTNNB1* knockdown in 3D culture (81.1% ± 6%, *p* = 0.006) versus Opti-MEM prepared siRNA (3.6% ± 42.1%, p = 0.93). We did however observe that the *CTNNB1* knockdown induced in 3D culture using 10% serum formed siRNA complexes was transient with significant knockdown demonstrated at 48 hours only by qRT-PCR. Consequently, we did not observe any phenotypic differences (data not shown) suggesting this approach is not suitable for sustained knockdown (where inducible shRNA systems are preferable). We hypothesized that serum-derived nucleases could be detrimental to siRNA integrity and so employed a nuclease-resistant siSTABLE^®^ (GE Dharmacon, Lafayette, CO) targeted to *CTNNB1* but still observed no significant β-catenin protein reduction (data not shown). As expected for 2D culture of SW1463 cells, Opti-MEM formed siRNA was more efficient for *CTNNB1* knockdown (86.1% ± 1.3%, *p* < 0.0001) versus 10% serum (28.3% ± 31.7%, *p* = 0.26) (Fig. 1E). We ruled out cell line specific phenomena for these observations since similar results were observed using 3D LS174T tumour spheroids (Fig. 2A and B). We also confirmed that these results were not transfection reagent-specific since siRNA complexes formed with Lipofectamine® 2000 reagent and Opti-MEM were also inhibited from entering LS174T tumour spheroids (Fig. 2C). We next investigated whether this method could be extended to the transfection of primary organoids. As observed in Fig. 3, organoids derived from the intestine of both *VilCre*^*ER*^ *Apc*^*fl/fl*^ *Kras*^*G12D/+*^ (Fig. 3A and B) and *wild type* mice (Fig. 3C and D) exhibited enhanced siRNA uptake when complexes were formed with the presence of 10% serum versus Opti-MEM.

**Figure 4:**
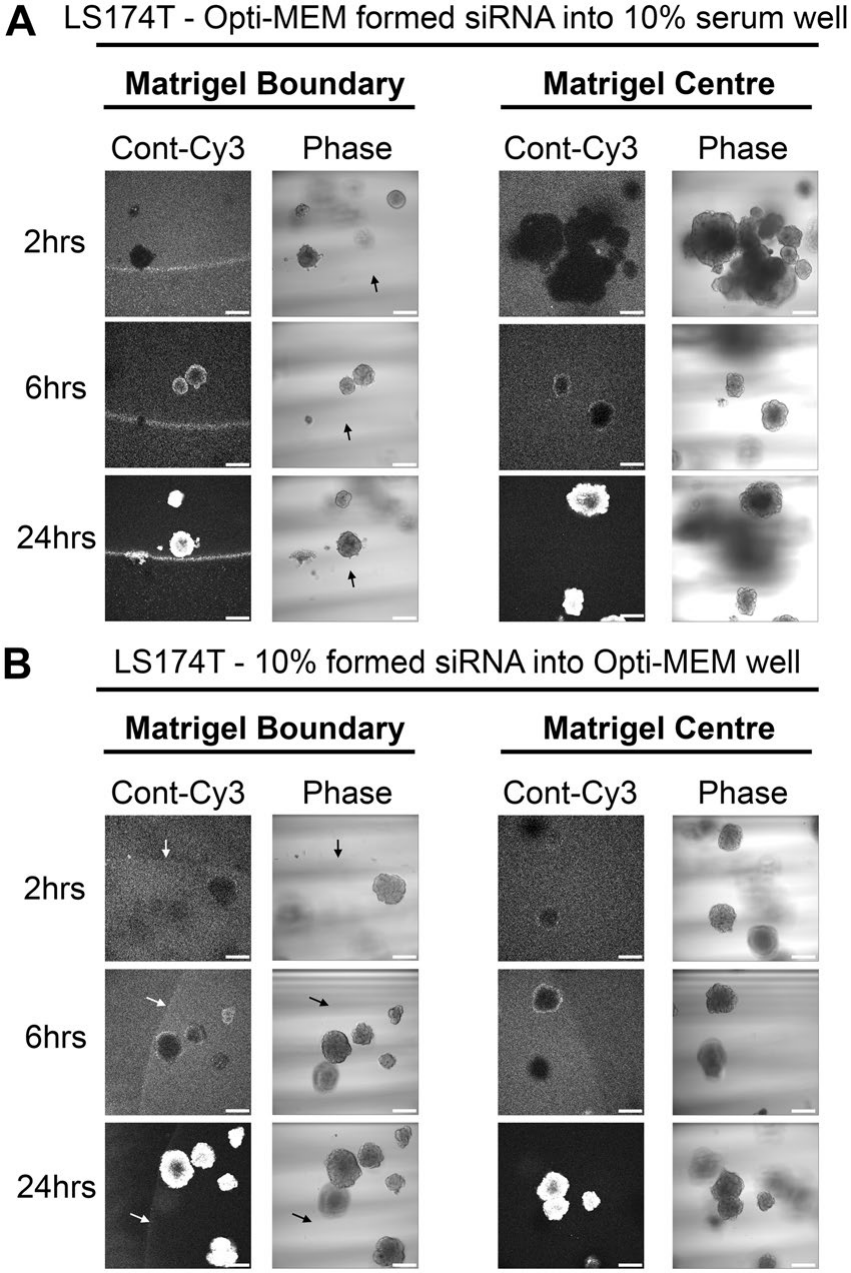
Matrigel and spheroid uptake of Opti-MEM-formed siRNA can be improved in the presence of 10% serum. (**A**) Representative confocal images of LS174T spheroids showing localization of control-Cy3 siRNA formed in 500 μl Opti-MEM using RNAiMax, but co-incubated in a well containing 1.5 mls DMEM containing 10% serum, at 2, 6 and 24 hours post transfection. (**B**) Representative confocal images of LS174T spheroids showing localization of control-Cy3 siRNA formed in 500 μl DMEM containing 10% serum using RNAiMax, but co-incubated in a well containing 1.5 mls Opti-MEM, at 2, 6 and 24 hours post transfection.

Finally, we examined whether the restricted uptake Opti-MEM-formed siRNA complexes could be relieved through co-incubation with 10% serum. Figure 4A demonstrates how the presence of 10% serum in the well greatly improved both the matrigel and spheroid uptake of Opti-MEM-formed siRNA complexes. At 24 hours however, there did remain a considerable proportion of fluorescence excluded at the matrigel boundary, suggesting siRNA complexes formed with Opti-MEM have a persistently decreased capacity for matrigel entry. We also performed the complementary experiment; does co-incubation with Opti-MEM restrict the uptake of serum-formed siRNA complexes. The presence of Opti-MEM appeared to delay full spheroid uptake of 10% serum formed siRNA until after 6 hours by the presence of Opti-MEM (Fig. 4B) but at 24 hours no fluorescence was observed at the matrigel boundary. Taken together, these data suggest siRNA formed in the presence of 10% serum has an enhanced capacity for matrigel penetration and spheroid/organoid uptake.

### 1–10% serum is optimal for siRNA uptake

**Figure 5:**
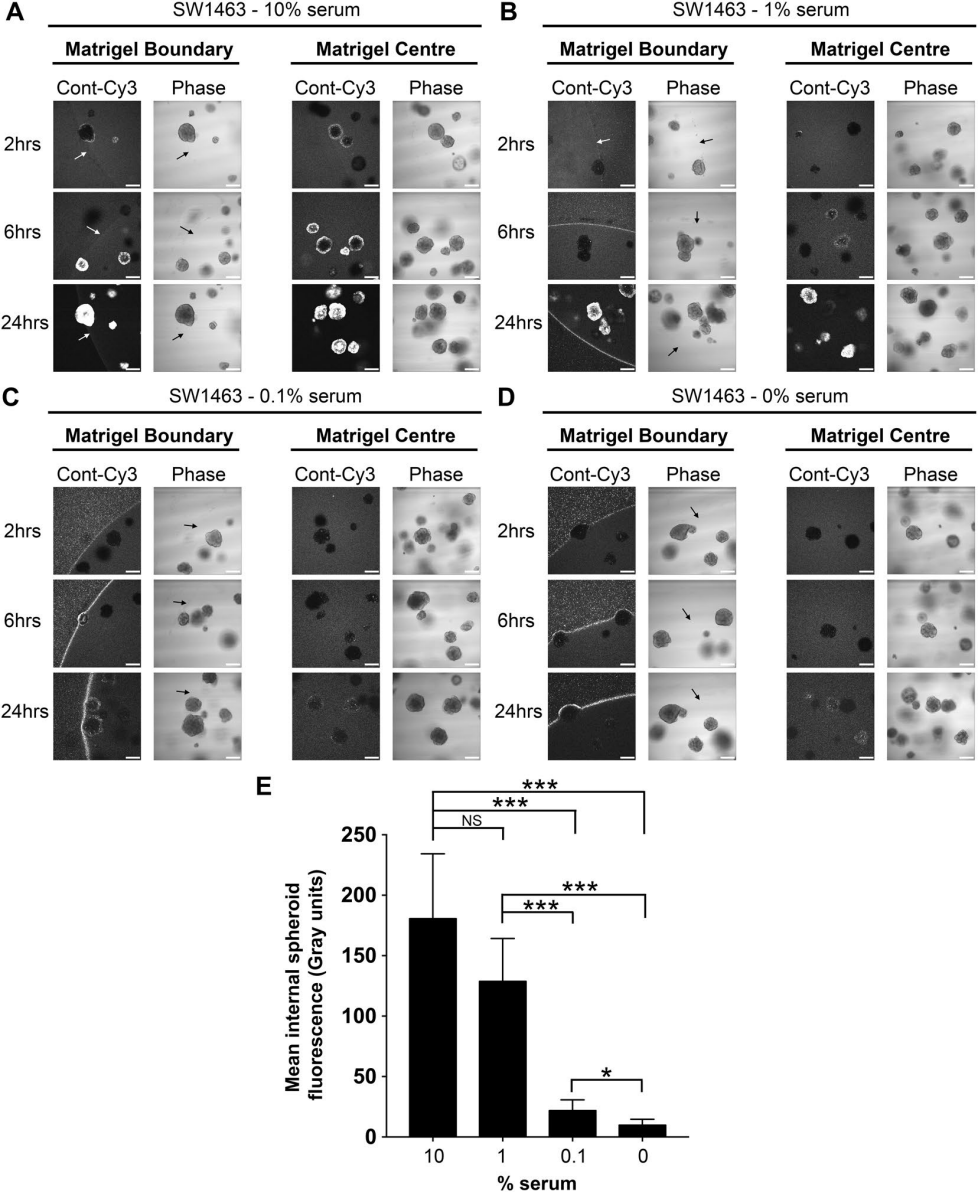
Optimal siRNA uptake by matrigel and spheroids occurs with a 1–10% serum concentration. Representative confocal images of SW1463 spheroids showing localization of control-Cy3 siRNA at 2, 6 and 24 hours post transfection with siRNA formed with (**A**) 10% (**B**) 1% (**C**) 0.1% or (**D**) 0% serum using RNAiMax. (**E**) Summary graph showing the mean internal spheroid fluorescence post transfection using siRNA formed with a dose-response of serum. Data represents mean ± 1 s.d (*n* = 3). Statistical significance is denoted by *P < 0.05 and ***P < 0.0001 (NS = not significant) as analyzed by a paired student’s t-test.

To confirm serum as the critical component of culture medium that facilitates siRNA uptake, and ascertain the optimal serum concentration, we formed siRNA complexes using a dose-response of serum from 10%, 1%, 0.1% and 0%. Figure 5A shows that siRNA prepared in DMEM containing 10% serum behaved as previous, with rapid uptake of fluorescence siRNA into the matrigel and spheroids in under 2 hours. As the concentration of serum dropped, a dose-dependent decrease in internal spheroid fluorescence occurred over 24 hours and fluorescence instead accumulated at the matrigel boundary (Fig. 5B–D). Figure 5E summarises how fluorescence internalization was optimal when siRNA complex formation was formed in the presence of 1–10% serum. At this point, we ruled out siRNA-specific phenomena since fluorescently tagged GAPDH-siRNA exhibited the same pattern of internalization during serum titration (Supplementary Figure S1). These data suggest a concentration of between 1–10% serum during siRNA complex formation is optimal for uptake by 3D spheroids.

### BSA alone can facilitate matrigel entry of siRNA but not spheroid uptake

**Figure 6:**
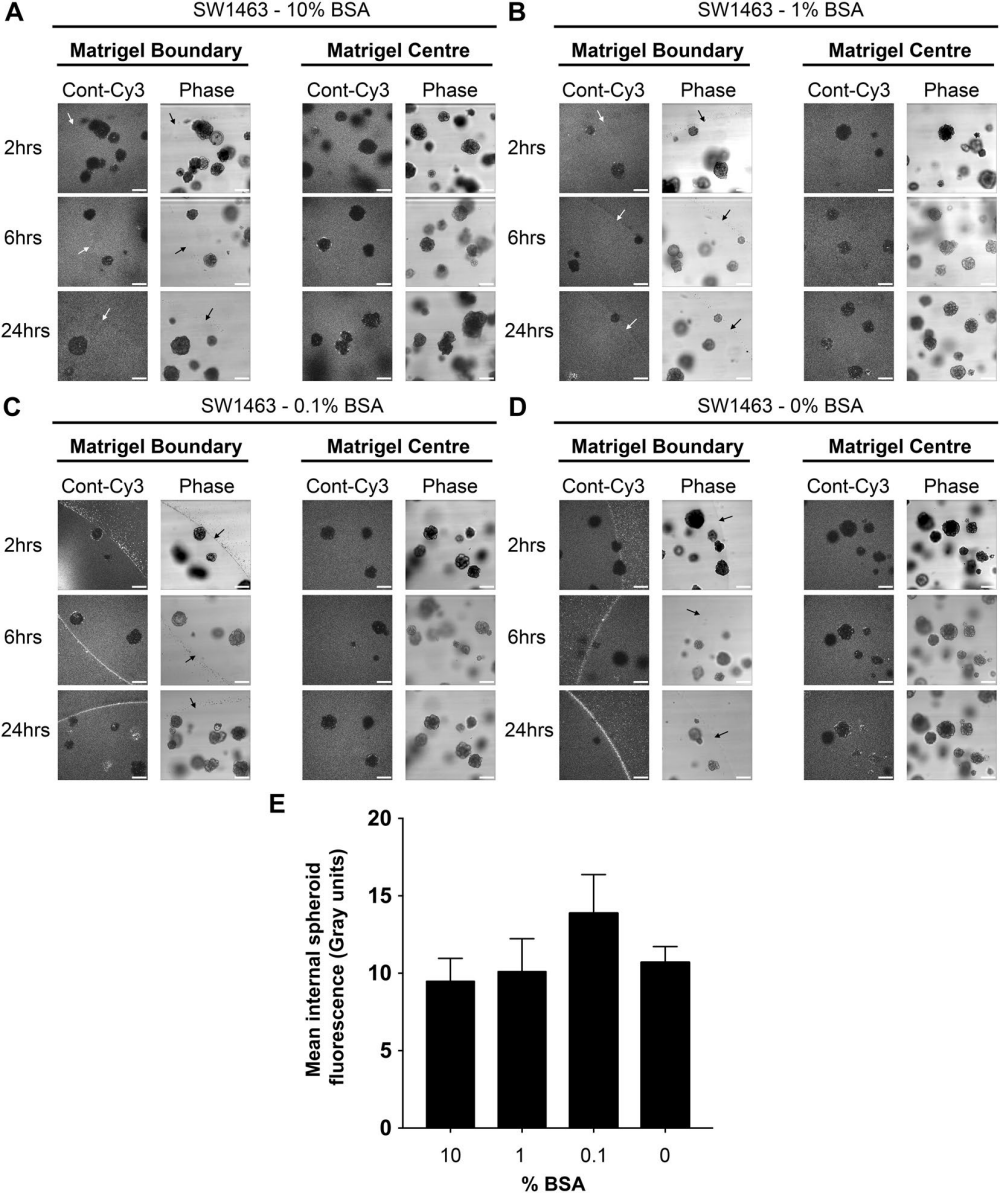
BSA can facilitate matrigel entry of siRNA, but not spheroid uptake. Representative confocal images of SW1463 spheroids showing localization of control-Cy3 siRNA at 2, 6 and 24 hours post transfection with siRNA formed in (**A**) 10% (**B**) 1% (**C**) 0.1% or (**D**) 0% BSA (no serum present) using RNAiMax. (**E**) Summary graph showing the mean internal spheroid fluorescence post transfection using siRNA formed with a dose-response of BSA. Data represents mean ± 1 s.d (*n* = 3).

Since albumin is a major protein within serum and has carrier properties^13,14^, we examined the efficiency of BSA alone (by dose response, no serum present) during siRNA complex formation for matrigel/spheroid penetration. Enhanced matrigel penetration by fluorescent siRNA could be seen with as little as 0.1% BSA, but interestingly no concentration of BSA was sufficient to promote internal spheroid fluorescence suggesting additional serum components are necessary for siRNA uptake (Fig. 6A–E).

### Serum molecules less than 10 kDa are dispensable for siRNA uptake

**Figure 7:**
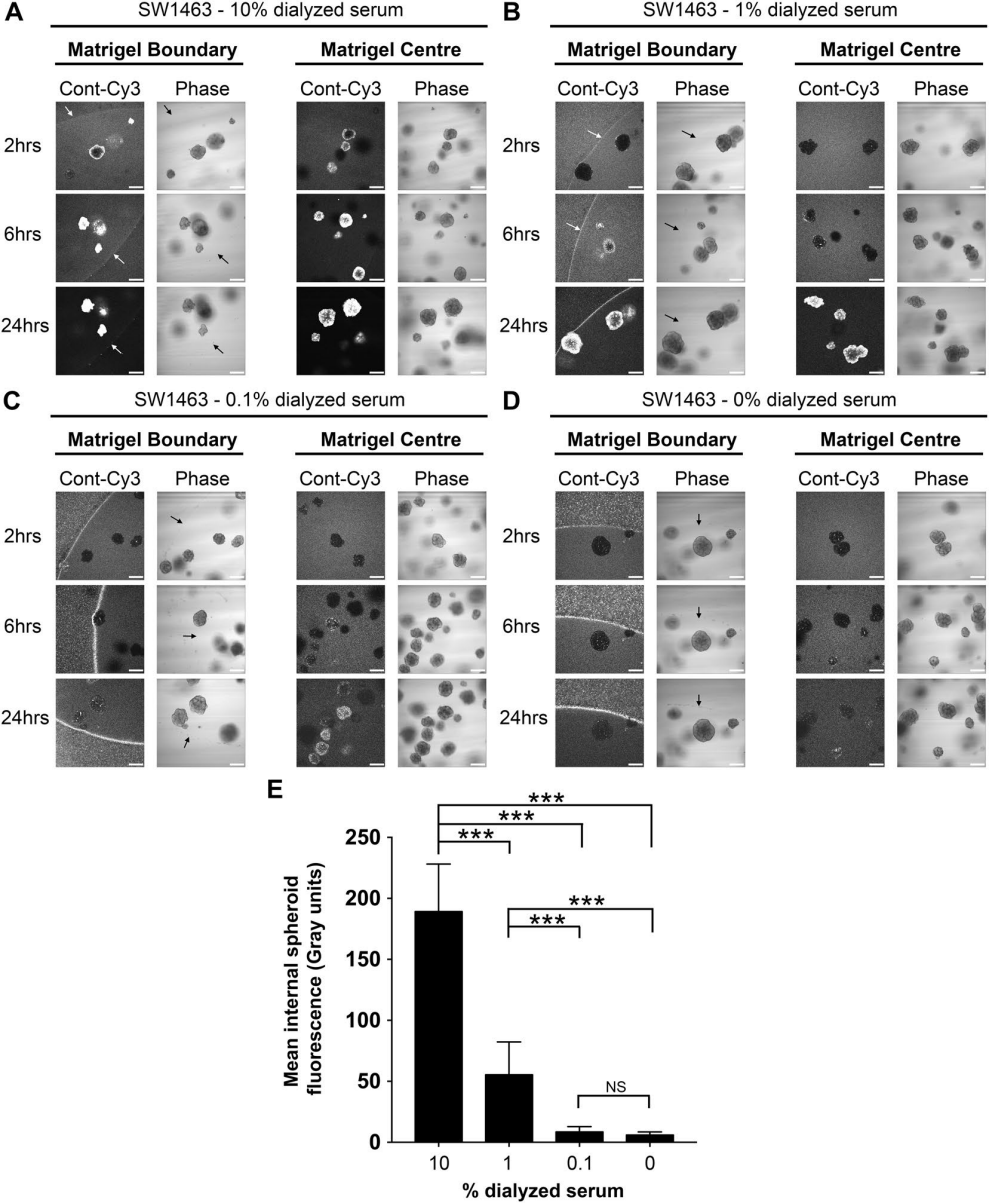
10 kDa dialyzed serum is also efficient for matrigel and spheroid uptake of siRNA. Representative confocal images of SW1463 spheroids showing localization of control-Cy3 siRNA at 2, 6 and 24 hours post transfection with siRNA formed with (**A**) 10% (**B**) 1% (**C**) 0.1% or (**D**) 0% dialyzed serum using RNAiMax. (**E**) Summary graph showing the mean internal spheroid fluorescence post transfection using siRNA formed with a dose-response of dialyzed serum. Data represents mean ± 1 s.d (*n* = 3). Statistical significance is denoted by ***P < 0.0001 (NS = not significant) as analyzed by a paired student’s t-test.

Primary organoid cultures are grown in strictly defined medium containing specialized growth factor supplements such as R-Spondin and Wnt3A^15,16^. Although supplemental growth factors are not required for the growth of SW1463 cells in 3D, we assessed the ability of siRNAs formed in basic 3D organoid medium (Advanced DMEM/F12 + NAC/B27/N2) to gain matrigel/spheroid entry. Similar to previous observations with reduced serum conditions, we found little siRNA uptake into matrigel and organoids at all time-points assessed (Supplemental Fig. 2). When culturing primary organoids, the presence of serum components such as small growth factors, cytokines and hormones maybe undesirable and so we assessed the ability of dialyzed serum to promote siRNA uptake. We observed that siRNA uptake by spheroids was similarly efficient when complex formation occurred in 10 kDa dialyzed serum (Fig. 7A–D). As summarized in Fig. 7E, internal spheroid fluorescence was again optimal between 1–10%, albeit a lower level of internal fluorescence was obtained at both the 1% and 0.1% concentrations of dialyzed serum (versus normal serum Fig. 5E). These data demonstrate that dialyzed serum was similarly efficient for matrigel penetration and/or spheroid uptake, and any soluble factors facilitating these processes are likely to be more than 10 kDa in molecular weight.

## Discussion

Gene silencing through siRNA is a well-established technique owing to its relative simplicity, convenience, cost-effectiveness and routine use in high-throughput screens. In spite of this, transient transfections of established organoids *in situ* with siRNA at a specific time-point during 3D culture is not routinely performed despite the demand, and previous attempts have been met with poor efficiency. Bhise and colleagues used nanoparticle technology to improve the transfection of gene delivery vectors into both 2D and 3D cultures^2^. Transfection efficiencies using commercially available reagents such as FuGENE HD and Lipofectamine 2000 were generally low, but even the best performing polymeric nanoparticles transfected 57 ± 6% of the cells in 2D culture and only 6 ± 1% of the cells in 3D culture, confirming the difficulty in targeting 3D cultures. Mellor *et al*., also used polyplexes, cationic lipids Lipofectamine and DCChol:DOPE, and the physical approach of tissue electroporation to optimise non-viral gene delivery to multicellular tumour spheroids (MCTS)^3^. Similar to us they used a colorectal cancer cell line (DLD-1) but used agarose instead of matrigel as the extra-cellular matrix. They observed spatially restricted transfection as fluorescent complexes could only penetrate the outer 3–5 proliferating cell layers, however it would be interesting to see if there was any accumulation of fluorescent complexes at the agarose boundary.

Observations in this study are consistent with the difficulty of performing gene silencing *in situ* with 3D cultures using siRNA:lipofection-based techniques. Fluorescent siRNA has revealed that reduced serum media like Opti-MEM is inefficient at delivering siRNA to cells in matrigel. Critically, it is the presence of serum (1–10%) during the siRNA complex formation stage that significantly enhances matrigel penetration and spheroid/organoid uptake. Although previous studies show localization of fluorescently tagged siRNA correlates with gene silencing in 2D culture systems^17–19^, we observed only a transient reduction in *CTNNB1* mRNA level (and consequently no detectable protein decrease by Western blotting; data not shown). Such transient gene knockdown renders protein reduction difficult to observe, particularly for the longer half-life β-catenin protein forms tethered at membrane complexes. Ly and colleagues recently trialled the use of a novel class of asymmetric, chemically stabilized, cholesterol-modified siRNAs referred to as sdrxRNAs®, which can enter cells and tissues without prior formulation^20^. Uptake and repression of gene expression by sdrxRNAs was efficient in 2D culture of HeLa cells, however, gene knockdown efficiency in 3D-matrigel based culture systems was not assessed. Data shown here suggests the addition of serum might improve the efficiency of this siRNA class for 3D culture use. The conditions assessed in this study, and their effect on fluorescent siRNA localization in 3D cultures, are summarized in Table 1 below.

**Table 1:** Summary of all conditions used to prepare siRNA and their subsequent effect upon siRNA uptake efficiency in 3D culture.

siRNA complexes formed in:	Matrigel penetration	Organoid uptake
Opti-MEM + Lipofectamine RNAiMAX	✗	✗
Opti-MEM + Lipofectamine 2000	✗	✗
Opti-MEM formed siRNA into well containing 10% serum	✓✓	✓✓
10% serum formed siRNA into well containing Opti-MEM	✓✓	✓✓
10% serum	✓✓	✓✓
1% serum	✓	✓
0.1% serum	✗	✗
10% BSA	✓	✗
1% BSA	✓	✗
0.1% BSA	✗	✗
DMEM only	✗	✗
3D organoid medium	✗	✗
10% dialyzed serum	✓✓	✓✓
1% dialyzed serum	✓	✓
0.1% dialyzed serum	✗	✗

The stark contrast between 2D and 3D transfection efficiencies (Fig. 1) highlight the key differences that exist during transfection of 2D versus 3D culture model systems. Rieux and co-workers assessed gene delivery using lipoplexes either encapsulated within or seeded onto hydrogel^5^. They found that the transgene expression increased over 11 days of culture but was significantly higher in 2D compared with 3D cultures. Interestingly, data from encapsulated complexes showed that the hydrogel component fibrin interacts with and sequesters the lipoplexes thus limiting cellular internalization and causing a delay in expression. Matrigel does not contain fibrin to our knowledge, but it does raise the prospect of other gel components sequestering siRNA complexes at the matrigel surface. Our BSA data show that even when the siRNA complexes gain matrigel entry they are not taken up by the spheroids, implying other serum factors promote this process. Marked differences in gene expression have already been noted between 2D and 3D systems^21–23^, and it would be interesting to see if proteins critical for siRNA uptake, such as connexin-43 (Cx43)^24^, are differentially expressed between 2D and 3D cultures. Finally, it may be useful to laboratories that use primary human/mouse organoids that dialyzed serum (with 10 kDa threshold) is works well for siRNA uptake since this will limit the presence of undesirable serum-derived small molecules such as growth factors, cytokines and hormones in carefully defined media.

Our study has increased understanding of why gene silencing studies *in situ* using siRNA with lipofection-based methods has proved ineffective in 3D culture to date. Moreover, we have been able to dramatically increase the efficiency of siRNA penetration through matrigel and into spheroid culture through the addition of serum during the transfection procedure. These developments will be useful for experimental approaches that require transient gene silencing in established 3D cultures and can be developed further to improve efficiency.

## Methods

### Cell culture

LS174T and SW1463 cells were seeded at approximately 500 single cells per well (24-well plate) into 50 μl growth factor reduced and phenol red-free matrigel (Becton Dickinson, Oxford, UK). 3D tumour spheroids were maintained in Advanced Dulbecco’s Modified Eagle’s medium (DMEM)/F12 (Gibco, Paisley, UK), 2 mM glutamine, 10 mM HEPES, 1 mM N-acetyl-cysteine, 0.1% BSA (Sigma-Aldrich, Dorset, UK), penicillin (100 U/ml), streptomycin (100 U/ml), 1 × N2, and 1 × B27 supplements (Invitrogen, Paisley, UK). Primary intestinal organoids derived from wild type and *VilCre*^*ER*^ *Apc*^*fl/fl*^ *Kras*^*G12D/+*^ mice were a kind gift from the Sansom laboratory (Beatson Institute, Glasgow, UK) and were maintained as previously described^15^. For 2D experiments, SW1463 were maintained as previously described for carcinomas in this laboratory^25^.

### siRNA transfection

Following 12 days of 3D culture, tumour spheroids were transfected with siRNA. For imaging experiments, organoids received either 25 nM Ambion® *Silencer*® Cy™3-labeled Negative Control or *Silencer*® Cy™3-labeled GAPDH siRNA (Invitrogen). For mRNA assessment, spheroids received either 25 nM *CTNNB1* or non-targeting control siRNA (unlabelled) as previously described^26^. siRNA complexes were formed using either Lipofectamine 2000 or RNAiMAX (Invitrogen), in either Opti-MEM (reduced serum) or DMEM (Gibco) containing either 10% normal/dialyzed FBS (Gibco). In all conditions antibiotics were omitted as recommended by the manufacturer to preserve viability during the transfection process. 500 μl of formed siRNA complex medium was then bathed over 50 μl matrigel contained within a single well of a 24-well plate overnight and replaced the following morning with normal spheroid/organoid medium as outlined above.

### Confocal laser scanning microscopy

Cy3 immunofluorescence in tumor spheroids/primary organoids was analyzed *in situ* at 2/6/24 hours post transfection using a Leica DM I6000 inverted epifluorescence microscope (Leica, Buckinghamshire, UK), with the resonant scanning head of a SP5-AOBS confocal laser microscope (Leica) and a x10 dry objective as previously described^27^. Images of the matrigel boundary were captured and Z-stacks were acquired through spheroids/organoids at their widest diameter to ensure a full cross-sectional view of the structure. Internal spheroid fluorescence was calculated by drawing regions of interest (ROI) around the entire circumference of the spheroid followed by measurement of mean Cy3 channel fluorescence intensity within this designated area using LAS AF software v2.6.0 (Leica). TIFFs were generated using Volocity confocal software v6.3.0 (Perkin-Elmer, Waltham, MA, USA) and post-acquisition processing performed using Photoshop CS6 v13.0 (Adobe, Berkshire, UK) with Cy3 channel levels adjusted (equal changes applied across the entire figure).

### RNA extraction from 3D culture and qRT-PCR

48 hours proceeding siRNA transfection of organoids, 3D culture medium was aspirated and matrigel scaffolds washed with ice cold PBS. Following wash, 500 μl of Tri-reagent® (Sigma-Aldrich) was added to each culture and incubated for 5 minutes on ice. RNA was purified from Tri-reagent® and *CTNNB1* mRNA assessed by qRT-PCR performed as previously described^26^.

### Statistics

All statistical analyses were performed using GraphPad Prism v7.0 (GraphPad Software, Inc., San Diego, CA). Significance of difference was assessed using a one-sample or students t test with significance defined at *P* < 0.05.

## Electronic supplementary material

Supplementary figures

## References

[1] Huch M, Koo BK (2015). Modeling mouse and human development using organoid cultures. Development.

[2] Bhise NS (2010). The relationship between terminal functionalization and molecular weight of a gene delivery polymer and transfection efficacy in mammary epithelial 2-D cultures and 3-D organotypic cultures. Biomaterials.

[3] Mellor HR (2006). Optimising non-viral gene delivery in a tumour spheroid model. J Gene Med.

[4] Oishi M (2007). Enhanced growth inhibition of hepatic multicellular tumor spheroids by lactosylated poly(ethylene glycol)-siRNA conjugate formulated in PEGylated polyplexes. ChemMedChem.

[5] des Rieux A, Shikanov A, Shea LD (2009). Fibrin hydrogels for non-viral vector delivery *in vitro*. J Control Release.

[6] Wang N (2014). Adenovirus-mediated efficient gene transfer into cultured three-dimensional organoids. PloS one.

[7] Koo BK, Sasselli V, Clevers H (2013). Retroviral gene expression control in primary organoid cultures. Current protocols in stem cell biology.

[8] Van Lidth de Jeude, J. F., Vermeulen, J. L., Montenegro-Miranda, P. S., Van den Brink, G. R. & Heijmans, J. A protocol for lentiviral transduction and downstream analysis of intestinal organoids. *Journal of visualized experiments: JoVE*, 10.3791/52531 (2015). 10.3791/52531 PMC4541580 25938265

[9] Matano M (2015). Modeling colorectal cancer using CRISPR-Cas9-mediated engineering of human intestinal organoids. Nature medicine.

[10] Schwank G (2013). Functional repair of CFTR by CRISPR/Cas9 in intestinal stem cell organoids of cystic fibrosis patients. Cell stem cell.

[11] Zhang Q (2015). Commensal bacteria direct selective cargo sorting to promote symbiosis. Nature immunology.

[12] Zoldan J (2011). Directing human embryonic stem cell differentiation by non-viral delivery of siRNA in 3D culture. Biomaterials.

[13] Baker ME (2002). Albumin, steroid hormones and the origin of vertebrates. J Endocrinol.

[14] Fasano M (2005). The extraordinary ligand binding properties of human serum albumin. IUBMB Life.

[15] Sato T (2009). Single Lgr5 stem cells build crypt-villus structures *in vitro* without a mesenchymal niche. Nature.

[16] Sato T (2011). Long-term expansion of epithelial organoids from human colon, adenoma, adenocarcinoma, and Barrett’s epithelium. Gastroenterology.

[17] Harborth J (2003). Sequence, chemical, and structural variation of small interfering RNAs and short hairpin RNAs and the effect on mammalian gene silencing. Antisense Nucleic Acid Drug Dev.

[18] Wittrup A (2015). Visualizing lipid-formulated siRNA release from endosomes and target gene knockdown. Nature biotechnology.

[19] Chiu YL, Ali A, Chu CY, Cao H, Rana TM (2004). Visualizing a correlation between siRNA localization, cellular uptake, and RNAi in living cells. Chem Biol.

[20] Ly S (2017). Visualization of self-delivering hydrophobically modified siRNA cellular internalization. Nucleic acids research.

[21] Zschenker O, Streichert T, Hehlgans S, Cordes N (2012). Genome-wide gene expression analysis in cancer cells reveals 3D growth to affect ECM and processes associated with cell adhesion but not DNA repair. PloS one.

[22] Price KJ (2012). Matrigel basement membrane matrix influences expression of microRNAs in cancer cell lines. Biochemical and biophysical research communications.

[23] Edmondson R, Broglie JJ, Adcock AF, Yang L (2014). Three-dimensional cell culture systems and their applications in drug discovery and cell-based biosensors. Assay Drug Dev Technol.

[24] Brink PR, Valiunas V, Gordon C, Rosen MR, Cohen IS (2012). Can gap junctions deliver?. Biochimica et biophysica acta.

[25] Moore AE (2009). HGF/Met signalling promotes PGE(2) biogenesis via regulation of COX-2 and 15-PGDH expression in colorectal cancer cells. Carcinogenesis.

[26] Petherick KJ (2013). Autolysosomal beta-catenin degradation regulates Wnt-autophagy-p62 crosstalk. The EMBO journal.

[27] Morgan RG (2015). Nutrient stress alters the glycosylation status of LGR5 resulting in reduced protein stability and membrane localization in colorectal tumour cells: implications for targeting cancer stem cells. British journal of cancer.

