# Supplementary material for: Optimized delivery of siRNA into 3D tumor spheroid cultures in situ
Source: Sci Rep. 2018 May 21;8:7952. doi: 10.1038/s41598-018-26253-3 (PMC5962539; doi:10.1038/s41598-018-26253-3)
Supplement: Supplementary file 1 — Supplementary figures [file 41598_2018_26253_MOESM1_ESM.docx]

**Article title:** Optimized delivery of siRNA into 3D tumor spheroid cultures *in situ*

**Running title:** *In situ* delivery of siRNA into 3D culture

**Article type:** Biological techniques

**Authors/Affiliations:** Morgan RG^1,2,†,^*, Chambers A^1,†^, Legge DN^1^, Coles SJ^3^, Greenhough A^1,‡,^* and Williams AC^1,‡^

^1^ School of Cellular and Molecular Medicine, University of Bristol, Biomedical Sciences Building, University Walk, Bristol, BS8 1TD, UK.

^2^ School of Life Sciences, University of Sussex, Brighton, BN1 9QG, UK.

^3^ Institute of Science and the Environment, University of Worcester, Worcester, WR2 6AJ, UK.

^†‡^ These authors contributed equally to the work

***Joint corresponding authors:**

Dr. Rhys Morgan, School of Cellular and Molecular Medicine, Biomedical Sciences Building, University Walk, University of Bristol, BS8 1TD, UK (Current).

School of Life Sciences, University of Sussex, Brighton, BN1 9QG, UK (Future).

Email: rhys.morgan@bristol.ac.uk

Tel: 01173312023

Dr. Alexander Greenhough, School of Cellular and Molecular Medicine, University of Bristol, Biomedical Sciences Building, University Walk, Bristol BS8 1TD, UK.

Email: a.greenhough@bristol.ac.uk

Tel: +44 117 331 2044

**Supplementary Figures**


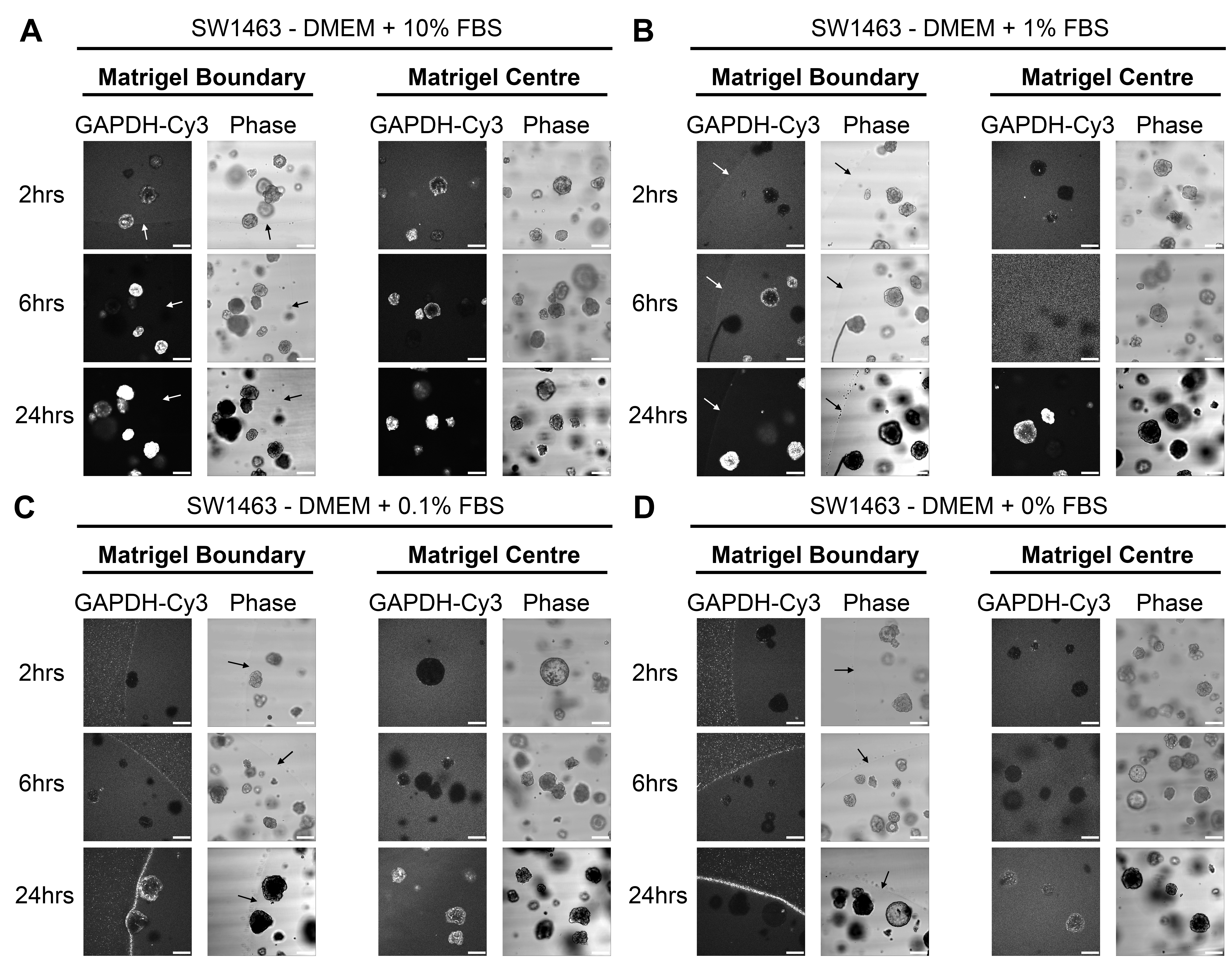


**Supplemental Figure S1 – The exclusion of fluorescent siRNA complexes from matrigel and organoids is not siRNA specific.** Representative confocal images of SW1463 organoids showing localisation of GAPDH-Cy3 siRNA 2, 6 and 24 hours post transfection with complexes formed in DMEM + A) 10% B) 1% C) 0.1% or d) 0% FBS. The GAPDH-Cy3 siRNA localises identically to the Control-Cy3 siRNA in response to FBS titration. White scale bar indicates 250μM and white/black arrows indicate the location of the matrigel boundary.


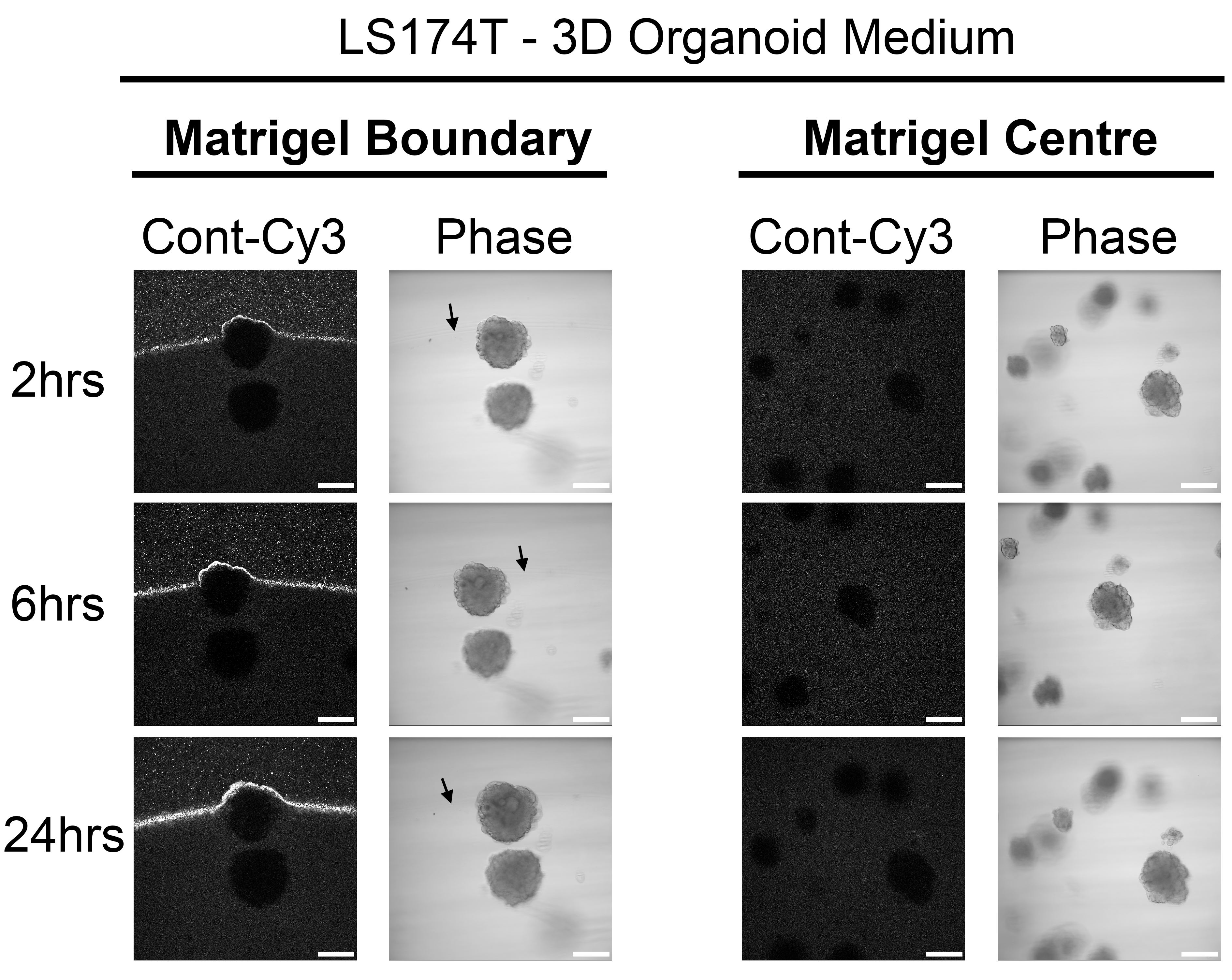


**Supplemental Figure S2 – SiRNAs formed in basic 3D organoid medium do not gain matrigel/spheroid entry.** Representative confocal images of LS174T spheroids showing localisation of control-Cy3 siRNA 2, 6 and 24 hours post transfection with complexes formed in basic 3D organoid medium (serum absent).
